# Demonstrative Reference and Semantic Space: A Large-Scale Demonstrative Choice Task Study

**DOI:** 10.3389/fpsyg.2020.00629

**Published:** 2020-04-07

**Authors:** Roberta Rocca, Mikkel Wallentin

**Affiliations:** ^1^Department of Linguistics, Cognitive Science and Semiotics, School of Communication and Culture, Aarhus University, Aarhus, Denmark; ^2^Interacting Minds Centre, Aarhus University, Aarhus, Denmark; ^3^Psychoinformatics Lab, Department of Psychology, The University of Texas at Austin, Austin, TX, United States; ^4^Center of Functionally Integrative Neuroscience, Aarhus University Hospital, Aarhus, Denmark

**Keywords:** language, semantics, spatial demonstratives, manipulability, the Demonstrative Choice Task

## Abstract

Spatial demonstratives (words like *this* and *that*) have been thought to primarily be used for carving up space into a peripersonal and extrapersonal domain. However, when given a noun out of context and asked to couple it with a demonstrative, speakers tend to choose *this* for words denoting manipulable objects (small, harmless, and inanimate), while non-manipulable objects (large, harmful, and animate) are more likely to be coupled with *that*. Here, we extend these findings using the Demonstrative Choice Task (DCT) procedure and map demonstrative use along a wide spectrum of semantic features. We conducted a large-scale (*N* = 2197) DCT experiment eliciting demonstratives for 506 words, rated across 65 + 11 perceptually and cognitively relevant semantic dimensions. We replicated the finding that demonstrative choice is influenced by object manipulability. Demonstrative choice was furthermore found to be related to a set of additional semantic factors, including valence, arousal, loudness, motion, time and more generally, the self. Importantly, demonstrative choices were highly structured across participants, as shown by a strong correlation detected in a split-sample comparison of by-word demonstrative choices. We argue that the DCT may be used to map a generalized semantic space anchored in the *self* of the speaker, the self being an extension of the body beyond physical space into a multidimensional semantic space.

## Introduction

Spatial demonstratives are one of the central ways in which language can be used to coordinate attention and enable social interaction. Words like the pronominal and adnominal forms *this* and *that*, or the adverbs *here* and *there* are among the few undisputed language universals (Diessel, 1999; Kemmerer, 1999). Demonstratives are developmental (Capirci et al., 1996) and evolutionary (Diessel, 2006, 2013; Pagel et al., 2013) cornerstones of language, and are among the most frequent words in the lexicon (Leech et al., 2014; Levinson, 2018).

Demonstratives are *deictic* expressions (from Greek *deixis*, “demonstration and indication”). They can in principle be used to indicate *any* object, and their meaning depends on the context of utterance (Levinson, 1983; Diessel, 1999). Identifying their referent in conversation hinges on the availability of information in the perceptual context (which objects are available), multimodal cues, such as pointing or gaze cuing (Cooperrider, 2016), expectations, i.e., what the speaker may *intend* to refer to (Levinson, 1983; Clark, 1996) and cues provided by the use of specific demonstrative forms (e.g., a proximal *this* vs a distal *that*).

We primarily use the proximal demonstrative (*this*) to refer to objects within manual reach (Coventry et al., 2008), but demonstratives are also used to establish contrasts in conceptual space, where meaning may be negotiated in the absence of visible objects and interlocutors. Experimentally, the use of specific demonstrative forms has been found to reveal information about the speaker’s relationship to the referenced object (e.g., ownership, familiarity; see Coventry et al., 2008, 2014; Rocca et al., 2019b) and about the conversational situation (Peeters and Özyürek, 2016; Rocca et al., 2019c). More generally, demonstratives may signal information about the functional status of the object and its affordances for interaction with respect to the speaker and/or the dyad (Jungbluth, 2003). In line with this, listening to demonstratives embedded in a dialogue has been shown to yield activation in the brain’s dorsal parietal cortices, suggesting a link between demonstrative use and where/how processing pathways (Rocca et al., 2019a). These findings show that demonstratives serve a fundamental role in linking language with non-linguistic cognition in order to guide joint attention during communication (Diessel, 2006).

In a recent study (Rocca et al., 2019b), we introduced the *Demonstrative Choice Task* (*DCT*), a new experimental paradigm where participants are asked to match nouns (e.g., *apple* or *tiger*) with a demonstrative (i.e., *this* or *that*) without any further context. Across three languages, we found that participants consistently use the distal demonstrative (*this*) for a word like *apple*, whereas they consistently choose *that* for a word like *tiger*. This effect was interpreted to be related to the inferred manipulability of the object, a compound metric combining perceptual (size), psychological (harmfulness), and semantic dimensions of the object. This is in line with research suggesting that demonstratives are interconnected with kinematic planning (Bonfiglioli et al., 2009; Rocca et al., 2018; Caldano and Coventry, 2019) and interactional affordances (Rocca et al., 2019c), rather than being mere distance indicators.

In this experiment, we further validated the DCT and explored whether semantic dimensions other than manipulability affect how speakers couple demonstratives and content words in the absence of context. First, we attempted to establish whether the distribution of demonstrative choices for particular words (i.e., how often a word is coupled with either *this* or *that*) are reproducible across a large set of words. Secondly, we aimed to replicate our previous finding that word meaning related to manipulability affects demonstrative choice. Thirdly, we tested if additional semantic domains have an influence on demonstrative choice, thus providing a comprehensive characterization of the relationship between semantics and demonstrative use. Lastly, we trained a classifier to investigate the degree to which individual trial level choices of *this* or *that* for particular words can be predicted by word semantics.

Demonstrative use depends on the establishment of an “origio,” serving as the frame of reference from which an utterance is constructed (Bühler, 1934/2011; Diessel, 2014). The semantic interpretation of *here* and *this* etc. thus presupposes a coordinate system anchored by some entity, usually the speaker’s body. However, we also know that spatial demonstratives can be used to denote non-spatial semantic features, such as time (e.g., *this time*), events (*this event*), emotions (*this emotion*), phenomenology (*this experience*), and abstract notions (*this abstraction*), that have no clear spatial anchoring. More generally, as noted by Bühler (1934/2011), deictic reference can be used in an imagination-oriented fashion (“deixis am Phantasma”), i.e., to refer to non-spatial entities such as discourse elements (anaphoric use), memories, imagined scenes, or other products of “constructive phantasy.”

Following this line of reasoning, we speculate that, when demonstratives are used to denote referents not immediately available in perceptual space, the proximal/distal distinction is anchored on a reference frame centered on the speaker’s *self*. The notion of self *includes* the speaker’s body but extends beyond the body to include multiple semantic dimension such as temporality (i.e., discourse markers such as anaphora), emotions, phenomenology, and social embeddedness (see Hanks, 2009; Stukenbrock, 2014 for similar suggestions). When non-spatial semantic entities are referred to, the interpretation of the proximal/distal distinction may thus be given by the position of the referent in a coordinate system consisting of psychological (e.g., familiarity and affect), semantic, and imaginative dimensions, anchored by the speaker’s position within that space.

In this study, we investigated demonstrative use in the latter scenario. We elicited demonstratives by presenting participants with concrete content words. No further context was provided. The words were rated along a comprehensive set of perceptually and psychologically relevant semantic dimensions (Binder et al., 2016). We expected the position of words (and the referred entities) within this semantic hyperspace to influence participants’ choices of demonstratives. Not all aspects of an object’s semantics might be equally relevant when choosing between proximal and distal demonstrative referencing expressions, and some dimensions are unlikely to contribute at all.

Using the simple behavior elicited by the DCT, we attempted to find out which individual features in the included set of semantic dimensions have an influence on speakers’ choices for specific demonstrative forms, and to estimate the extent of such effects.

## Materials and Methods

### Participants

We conducted a large-scale DCT experiment using Qualtrics^1^ with participants recruited through the Prolific website^2^. A total of 2,197 native English-speakers participated (gender: 1,364 female, 819 male, and 13 other; age: 801 were 18–30 years, 693 were 30–40 years, 347 were 40–50 years, 244 were 50–60 years, and 111 were 60+ years). The study was approved by the Institutional Review Board at Aarhus University.

### Procedure

The study took on average 4 min to complete, and participants were rewarded with 0.42 GBP for participation. Participants were presented with 48 or 49 words, selected from a database of 535 words, which have been rated on 65 different semantic dimensions, comprising sensory, motor, spatial, temporal, affective, social, and cognitive experiences (Binder et al., 2016). The 535 words were divided into 11 subsets, and participants were presented with one such subset of words in a pseudorandomized manner. Similar to our previous experiment (Rocca et al., 2019b), participants were asked to couple each word with either the spatial demonstrative *this* or with *that* without further context. They were instructed to simply follow their intuition and choose the combination of demonstrative and word they thought fitted best.

### Materials

**FIGURE 1:**
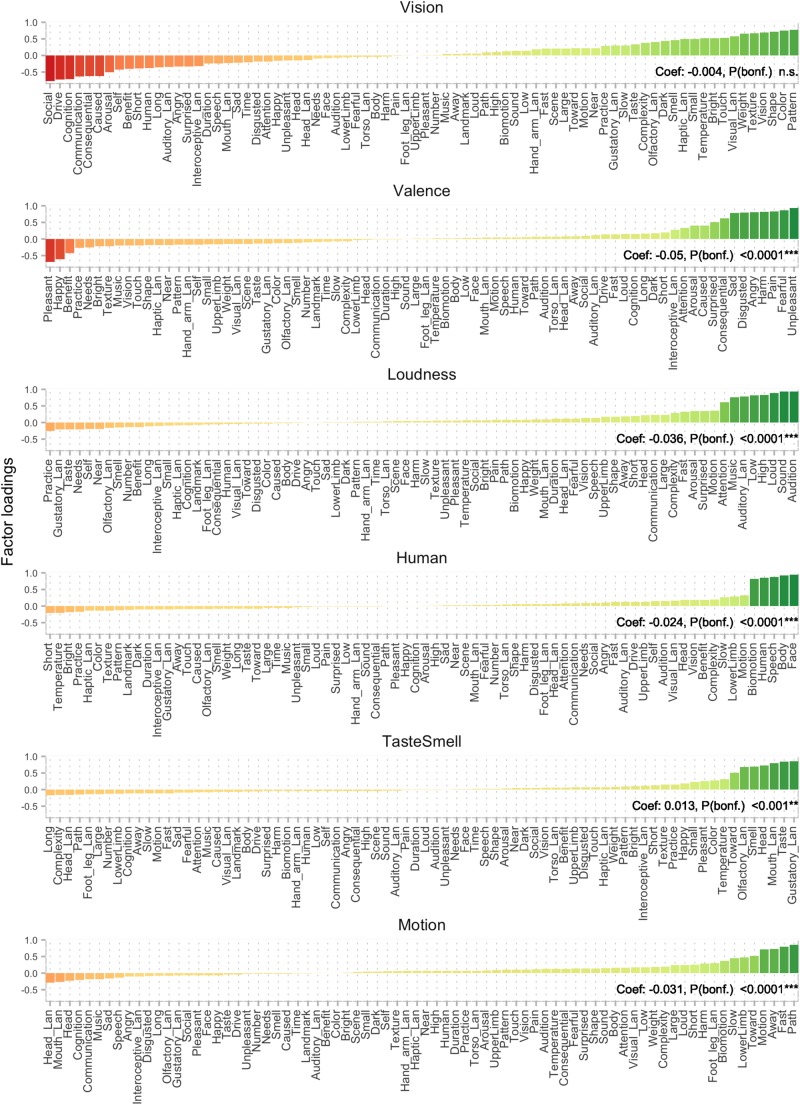
Factor analysis on a combination of Binder and Lancaster features resulted in 12 factors. Here, factors 1–6 are displayed (see Figure 2 for factors 7–12), with features ordered by loading. Factors are labeled by the authors. Coefficients reflect aggregate level regression results. A significant positive coefficient means that positive (green) sematic features are likely to elicit a proximal demonstrative, whereas features with negative (red) loadings tend to elicit distal demonstratives. When the coefficient is negative, the effect of the factor is reversed in the regression, i.e., features with positive loadings (green) are more likely to elicit distal demonstratives.

**FIGURE 2:**
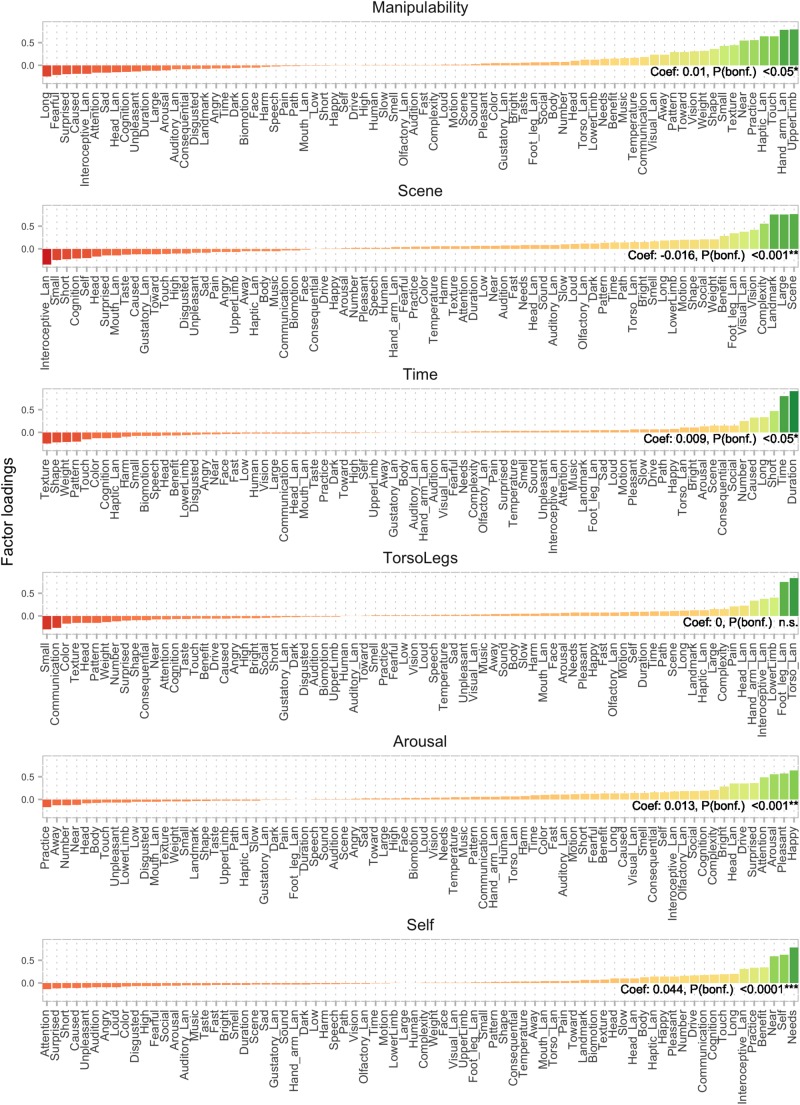
Factor analysis on a combination of Binder and Lancaster features resulted in 12 factors. Here, factors 7–12 are displayed, with features ordered by loading. Factor 7 (top panel) represents manipulability, which was hypothesized and found to explain demonstrative choice together with nine other semantic factors. Coefficients reflect aggregate level regression results. See Figure 1 for additional details.

The 65 semantic dimensions that words are rated along in the Binder dataset are: *Vision*, *Bright*, *Dark*, *Color*, *Pattern*, *Large*, *Small*, *Motion*, *Biomotion*, *Fast*, *Slow*, *Shape*, *Complexity*, *Face*, *Body*, *Touch*, *Temperature*, *Texture*, *Weight*, *Pain*, *Audition*, *Loud*, *Low*, *High*, *Sound*, *Music*, *Speech*, *Taste*, *Smell*, *Head*, *UpperLimb*, *LowerLimb*, *Practice*, *Landmark*, *Path*, *Scene*, *Near*, *Toward*, *Away*, *Number*, *Time*, *Duration*, *Long*, *Short*, *Caused*, *Consequential*, *Social*, *Human*, *Communication*, *Self*, *Cognition*, *Benefit*, *Harm*, *Pleasant*, *Unpleasant*, *Happy*, *Sad*, *Angry*, *Disgusted*, *Fearful*, *Surprised*, *Drive*, *Needs*, *Attention*, and *Arousal* (see Figures 1, 2 for illustrations of these features). The database is publicly available at: http://www.neuro.mcw.edu/representations/index.html, and the rationale for the choice of these exact features is that they represent “experiential phenomena for which there are likely to be corresponding distinguishable neural processors” (Binder et al., 2016). The notion that these features should have clearly defined neural underpinnings suggests that they are somehow important and representative for human cognition (see Binder et al., 2016 for further details).

One of the aims of the present work was to test the replicability of results from Rocca et al. (2019b), where manipulability is argued to play a role in demonstrative choice. The Binder et al. (2016) dataset does not provide an explicit manipulability dimension. We initially attempted to extract a proxy for manipulability applying principal component analysis and factor analysis to the Binder dimensions. However, we found no component that could straightforwardly be interpreted as manipulability. We therefore added to our feature set the Lancaster Sensorimotor Norms^3^. This dataset provides ratings along 11 sensorimotor features for a large body of words (Lynott et al., 2019). The 11 dimensions are the following (the affix *Lan* is appended to differentiate from features from the Binder dataset)*: Auditory_Lan*, *Gustatory_Lan*, *Haptic_Lan*, *Interoceptive_Lan*, *Olfactory_Lan*, *Visual_Lan*, *Foot_leg_Lan*, *Hand_arm_Lan*, *Head_Lan*, and *Mouth_Lan* (see Figures 1, 2 for illustrations of the features ordered according to semantic factors, obtained by factor analysis).

The overlap between the two databases included 506 out of the original 535 word, i.e., all words for which semantic ratings were available in both the Binder et al. (2016) and the Lancaster Sensorimotor Norms dataset. All subsequent analyses are conducted on this subset of the data, using the 65 + 11 semantic feature set. All feature ratings were standardized to make them comparable. Two Binder features contained missing ratings for particular words. These were imputed using the mean of all other words along that feature.

### Factor Analysis

We reduced the dimensionality of the semantic space using factor analysis. This was aimed at lowering the number of correlated regressors to be used in statistical analyses while preserving structural factors of the semantic space. To determine the number of latent factors, we used Horn’s parallel method (Horn, 1965), implemented in the psych package (Revelle, 2019) in R. This method compares the scree plot from the observed data with one made from random samples (randomized across rows) of the original data, and subtracts out the components that explain less variance than a comparable factor based on non-informative data [see analysis script (text footnote 3) for an illustration]. The estimated number of non-random factors in the semantic features using this procedure was 12. Factor analysis was conducted using ordinary least squares (OLS) to find the minimum residual (minres) solution. Orthogonal rotation (varimax) was applied. The cumulative proportion of variance of the semantic features explained by the 12 factors was 0.75.

Factors were labeled by the authors by inspecting the features yielding the highest factor loadings (see Figures 1, 2). The 12 factors and the proportion of the variance they explained in the original semantic space were: *Vision* (0.14), *Valence* (0.11), *Loudness* (0.09), *Human* (0.06), *Taste/Smell* (0.06), *Motion* (0.06), *Manipulability* (0.06), *Scene* (0.05), *Time* (0.03), *Torso/Legs* (0.03), *Arousal* (0.03), and *Self* (0.03) (see Figures 1, 2). It is important to note that these factors and the relative variance they explain do not necessarily reflect the general distribution in language or semantics, but only in the underlying sample of words and features present in the combined Binder and Lancaster databases. The ordering of the factors is therefore also partly specific to those words.

The 12 factors were used as predictors in two analyses (see below for details): (1) an aggregate-level linear regression analysis investigating the role of semantic dimensions in the distribution of demonstrative choices for words; (2) a logistic regression classifier investigating the degree to which trial by trial choices of *this* or *that* can be predicted by semantic factor scores of the experimental words.

### Inferential Aggregate Level Analyses

We first analyzed the data at an aggregate level, focusing on the overall proportion of proximal demonstratives chosen for each word as outcome variable.

The first aim of the analysis was to investigate the consistency in demonstrative choices across participants and words. We divided the data into two participant subsamples and calculated the proportion of proximal demonstratives chosen for each word in each sample. This yielded a vector of 506 proportion values (one per word) per participant sample. If participants’ choices of demonstrative forms for each word were random or inconsistent, we would expect the two vectors to be uncorrelated or only very weakly correlated. A strong correlation would speak in favor of participants’ coupling of demonstratives and words being highly structured and thus, at least to some extent, predictable.

Secondly, we conducted a linear regression analysis with the overall proportion of proximal demonstratives chosen for each word as dependent variable and the 12 factors as independent variables. This allowed to determine which (if any) semantic factors could be used to predict demonstrative choices.

### Trial-Level Classification Analysis

To examine the degree to which semantic factors could predict demonstrative choice at the single trial level, i.e., to determine how often word semantics could predict the choice of *this* and *that* on individual trials, we conducted a logistic regression classification analysis using the caret package in R. Individual trial data were initially divided into a training set (80%) and a test set (20%). The test set did not include any data from participants who were part of the training set.

The training set was used to conduct a logistic regression with 10-fold cross-validation. Again, we made sure that each fold in the cross-validation procedure did not contain data from participants that the data had been trained on.

The performance of the model was evaluated using a Monte Carlo permutation test (Ernst, 2004). Here, prediction performance is evaluated in terms of the probability of the observed prediction accuracy given the null. The null distribution of the prediction metric is obtained by randomly permuting the outcome labels, and fitting the model of interest to the permuted labels. To obtain the null distribution, we performed 1,000 permutations on all the data in the training set and obtained a probability value for our prediction score under the resulting distribution.

## Results

### Descriptive Results

The overall proportion of proximal/distal demonstratives in the data was 0.465/0.535 (standard deviation of proportion of proximal demonstratives across words: 0.114).

### Aggregate Level Results

**FIGURE 3:**
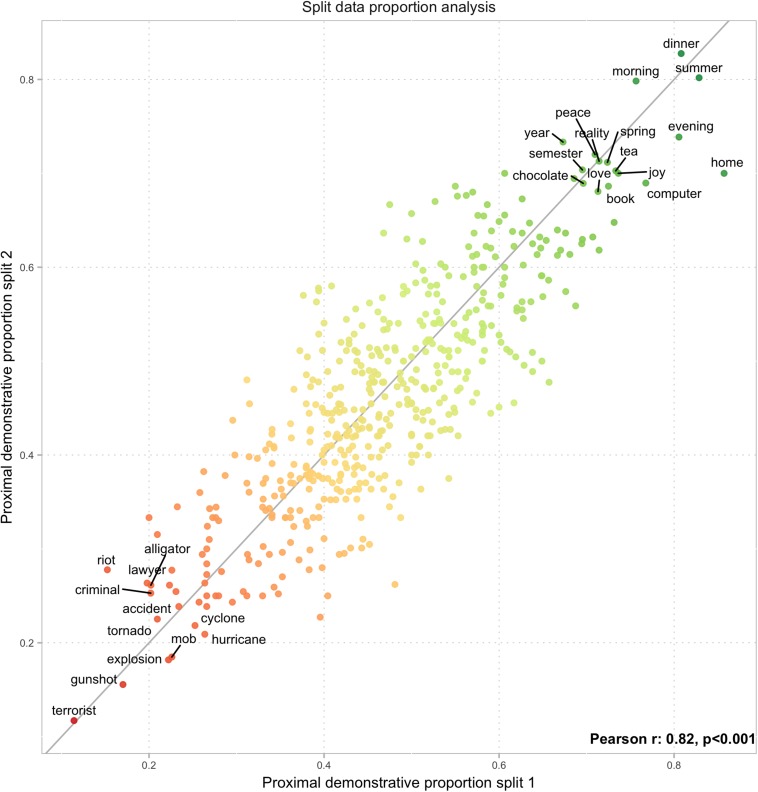
Aggregate proportion of proximal demonstratives in two data splits show a high degree of reproducibility (*r* = 0.82) in aggregate demonstrative choices for words across semantic categories. Words deviating more than two standard deviations from mean aggregate proportion are reproduced. Valence effect is clearly visible; negative effect of words denoting loudness as well as a positive effect of words relating to temporal events also seem to be visible.

In the split-sample reliability analysis, the proportion of proximal demonstratives was highly correlated across the two samples [*r* = 0.82, *t*(503) = 32.7, *p* < 0.0001; Figure 3], which speaks in favor of participants’ choices of demonstrative forms not being random.

The linear regression model with semantic factors (Figures 1, 2) as independent variables and overall proportion of proximal demonstratives and dependent variable was highly significant (adjusted *R*^2^ = 0.6018), indicating that the semantic factors explained variability in the distribution of proximal/distal demonstratives.

Out of the 12 semantic factors, 10 significantly contributed to the model (*p* < 0.05, Bonferroni corrected): *Valence* [*t*(493) = −15.6, *p* < 0.0001], *Loudness* [*t*(493) = −11.3, *p* < 0.0001], *Human* [*t*(493) = −7.4, *p* < 0.0001], *Taste/Smell* [*t*(493) = 4.0, *p* < 0.001], *Motion* [*t*(493) = −9.4, *p* < 0.0001], *Manipulability* [*t*(493) = 3.1, *p* < 0.05], *Scene* [*t*(493) = −4.5, *p* < 0.0001], *Time* [*t*(493) = 2.9, *p* < 0.05], *Arousal* [*t*(493) = −4.0, *p* < 0.001], and *Self* [*t*(493) = 13.0, *p* < 0.0001]. The factors *Vision* and *Torso/Legs* were non-significant (*p* > 0.05). Positive coefficients (see Figures 1, 2) and *t*-values indicate that the factor contributes positively to the choice of proximal demonstratives (i.e., elicits *this* more often), whereas negative coefficients and *t*-values indicate a negative contribution to the choice of proximal demonstratives (i.e., elicits *that* more often).

### Single Trial Level Results

The logistic regression model with semantic factors as dependent variables and individual trial choices of proximal/distal demonstratives as predictor led to a prediction accuracy of 57.61% on the training data and an accuracy of 58.40% on the test data (chance level 53.54% in the training set and 53.32% in the test set). All the 1,000 permutations in the null-distribution were lower than these values (range: 53.50–53.58%, based on the training set), indicating that the probability of the model belonging to a random distribution is <0.001.

Out of the 12 semantic factors, 10 significantly contributed to the model (*p* < 0.001, Bonferroni corrected): *Valence* (*z* = −28.323), *Loudness* (*z* = −20.152), *Human* (*z* = −12.946), *Taste/Smell* (*z* = 6.657), *Motion* (*z* = −17.105), *Manipulability* (*z* = 5.173), *Scene* (*z* = −9.671), *Time* (*z* = 6.082), *Arousal* (*z* = 7.213), and *Self* (*z* = 22.909). The factors *Vision* and *Torso/Legs* were non-significant (*p* > 0.05). The results thus closely mirror those from the aggregate level.

**FIGURE 4:**
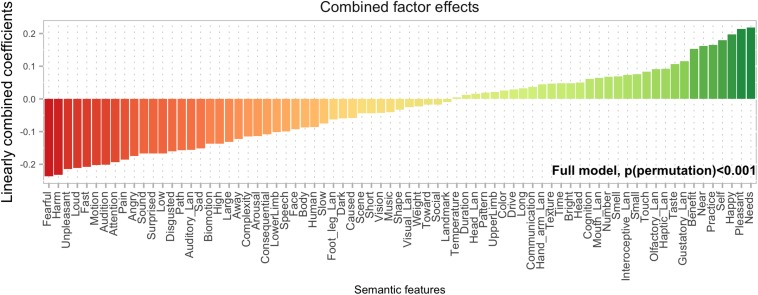
A linear combination of factor loadings and regression coefficients for the 10 significant components shows which semantic features drive demonstrative choice for proximal (green) and distal (red) demonstratives.

A linear combination of factor loadings and regression coefficients for the 10 significant components allows us to project the effects back into feature space (Figure 4). This shows how valence is an important driver of demonstrative choice, in combination with self-relatedness, proximity and features relevant for manipulability. Negative valence, motion, and loudness drive choices toward the distal demonstrative.

## Discussion

We documented that the DCT (a seemingly meaningless task) yields highly reproducible results. The proportion of proximal demonstratives for specific words in one randomly selected split of the data closely matched the proportion in the complementary participant sample (see Figure 3). This shows that the DCT is a reliable method for characterizing the relationship between demonstrative use and word meaning.

We additionally replicated the result (Rocca et al., 2019b) that affordances for manipulability predict the choice of proximal demonstratives. This effect, however, was found in a combination with nine additional semantic factors that also contributed to demonstrative choice.

On the face of it, the manipulability effect in the current experiment seems less pronounced than the one found in our previous study (Rocca et al., 2019b). The regression coefficient is smaller than several other factors (see Figures 1, 2), suggesting that the manipulability factor is not the main driver of semantic effects in this experiment. This is also clearly visible in Figure 4, where semantic features related to manipulability are overshadowed by those related to valence etc. However, manipulability can be more or less narrowly defined. In the previous study (Rocca et al., 2019b) manipulability was defined along three dimensions: “Can you move it with your hands?”, “Do you want to move it with your hands?” and “Will it let you move it with your hands?” Together, these dimensions yield a broad definition of manipulability that includes valence (“Do you want to move it…”) and animacy (“Will it let you move it…”). In the present experiment, the manipulability factor spans a narrower semantic space, more akin to “Can you move it…,” while leaving the other elements of the broader definition to, e.g., the *Valence* and to some degree the *Motion* factors. The present results thus provide evidence that demonstrative choice is affected by manipulability, even with this narrower definition of the term.

When combining the effects of semantic factors obtained via factor analysis and projecting them back into the original feature space, we find that features related to the experiential self (e.g., *Needs*, *Pleasant*, and *Happy*) dominate over features related to proximity and the physical self (e.g., *Near*, *Haptic_lan*). Whether this effect reflects a hierarchy presents outside the experiment or whether it is brought about by the format of the present experimental paradigm (where objects are not spatially available) remains to be investigated. Regardless, this study clearly shows that demonstrative choices signal an entity’s status along a wide array of semantic dimensions. Given the importance of communicating epistemic and emotional information, it may not be very surprising that demonstratives, being universal linguistic tools, can also be brought to work in these domains. Taking this line of thought a bit further, we hypothesize that demonstrative choices in the DCT, and perhaps in naturalistic demonstrative use (although this remains untested in this setup), can be taken as indices of the position of a referent relative to the speaker not only in a physical, but also in a *semantic* space. This builds on the idea that the speaker is the *origo* of the coordinate system against which demonstrative choices are evaluated both in physical and conceptual, psychological, and imaginary hyperspaces.

At the single trial level, we were able to predict 58.5% of the DCT trials in a test sample, based on the semantic profile of the word. The overall proportion of demonstratives was 46.5% for proximal and 53.5% for distal demonstratives, leading the null-distribution to be centered narrowly around 53.5%. A score of 58.5% correct classifications has to be measured both against the chance level of 53.5% and against the upper limit of predictability. The proportion of proximal demonstratives chosen for individual words differed from 50%, on average, with 9.5% (either above or below). Demonstrative choice for a single trial instantiation of a word which overall receives 50%/50% proximal/distal demonstratives can never be predicted above 50% based on information about the word itself, e.g., semantic factor scores. Given semantic scores for a certain word, a statistical model will end up always predicting the same demonstrative for this particular word, which can only be correct in 50% of the cases. Words that receive either a very low or a very high proportion of proximal demonstratives, on the other hand (e.g., *terrorist* or *summer* – see Figure 3), can theoretically be predicted with high accuracy. A good model predicting “proximal demonstrative” for a word that received, say, 70% proximal demonstratives, can yield 70% prediction accuracy for this word. The upper limit for classification thus becomes how far from 50% proximal demonstratives words are *on average*. If, across all words, the average absolute difference between the observed proportion of proximal demonstratives and the chance value of 50% is 9.5%, then the upper limit for predicting single trial choices based on semantics is 59.5%. With that in mind, a prediction rate of 58.5% is very close to ceiling for trial level predictions.

It is possible, however, that additional variables exist beyond word level semantics that systematically influence demonstrative choice and that these, if included in the models, would enable better predictions. To provide an example, it could be hypothesized that a 50%/50% distribution of proximal/distal demonstratives for a word could result from a particular subsample of participants always choosing the proximal demonstrative for this word, whereas another would never do so. If valence is a guiding principle, one could imagine that a word like *dog* would be given a proximal demonstrative by all dog lovers, whereas people who dislike dogs will use a distal form. This line of reasoning opens up to the possibility that the DCT could be used to probe individual differences among participants and that taking individual differences into account would raise the predictive power of the model. Demonstrative choice may thus be affected by the way preferences, experiences, and personality traits form our individual semantic landscape. If this is indeed the case, participants’ response patterns in the DCT could be used as a tool to predict individual differences in personality, a possibility which needs to be explored in further studies.

As a final note, the DCT provides consistent results on the effect of semantic factors in isolation, but demonstratives are most often used in exophoric contexts with an actual spatial configuration of speaker, referent, and addressee. These factors are key to shaping demonstrative use in naturalistic settings, and they are likely to interact with semantics, e.g., introducing contextual modulations of the effect of specific semantic dimensions. Further studies are needed to clarify to what extent and how semantics influences the choice of demonstratives in more contextualized situations where competing forces govern the selection. Experimentally assessing the relative role of these factors and their interactions may also inform computational models predicting demonstrative choice, a hitherto unexplored avenue in the field.

## Conclusion

In this study, we validated the use of DCT as a simple method to characterize the relationship between demonstrative use and semantic spaces. We have found that demonstrative choice is influenced by multiple semantic dimensions, including spatial, bodily, and emotional features, and we have showed that demonstratives might be consistently used to signal the relation between speakers and objects not only within physical space, but also in semantic hyperspaces. Further developments to the paradigm may increase the predictive power of the DCT as well as revealing new practical applications.

## Data Availability Statement

All data and scripts for analyses are available from Open Science Framework: https://osf.io/tqejb/.

## Ethics Statement

The studies involving human participants were reviewed and approved by the Aarhus University’s Research Ethics Committee. The patients/participants provided their written informed consent to participate in this study.

## Author Contributions

RR and MW devised the experiment, conducted the statistical analyses, wrote the manuscript together, and approved the manuscript before submission.

## Conflict of Interest

The authors declare that the research was conducted in the absence of any commercial or financial relationships that could be construed as a potential conflict of interest.

## Footnotes

1 http://qualtrics.com

2 http://prolific.ac

3 Available at https://osf.io/7emr6/
